# Definition and neurobiological underpinnings of treatment resistance in bipolar disorder

**DOI:** 10.1192/j.eurpsy.2025.106

**Published:** 2025-08-26

**Authors:** L. Jonsson

**Affiliations:** Department of Psychiatry and Neurochemistry, Institute of Neuroscience and Physiology at the Sahlgrenska Academy, University of Gothenburg, Gothenburg, Sweden

## Abstract

**Abstract:**

The pharmacological treatment strategies for bipolar disorders include both acute and maintenance treatments. However, a substantial number of patients do not respond sufficiently to pharmacotherapy. Thus far, there has been no standardized definition for treatment resistance in bipolar disorder. Studies have been using different definitions, possibly due to the clinical complexity including the episodic nature of bipolar disorder and various subtypes. The talk will discuss the definitions, neurobiological, and genetic aspects as well as ongoing initiatives to identify markers for treatment resistance in bipolar disorder.

**Disclosure of Interest:**

None Declared

